# 

**Published:** 1867-08

**Authors:** 


### ORIGINAL CONTRIBUTIONS.

ART. XXXI. Supplemental Report from the Committee of Practical Medicine. By T. L. Hewins, Loda, Ill. 449

ART. XXXII. Deformities of the Feet. By Julien S. Sherman, M.D., Chicago 465

ART. XXXIII. Olive Oil in Large Doses in Gall-Stone Disease. By Ira Hatch, M.D., Chicago 469

ART. XXXIV. A Few Observations Upon the Subject of Digestion. By R. Dexter, M.D., Chicago 472

ART. XXXV. Tape-Worm. (Tænia Solium.) By S. A. McWilliams, A.M., M.D., Chicago 474

ART. XXXVI. Report on the Prevalence of Disease in Chicago, for the Months of April, May and June, 1867. By N. S. Davis, M.D. (Read to the Chicago Medical Society.) 475

### PROCEEDINGS OF SOCIETIES.

Morgan County Medical Society 478
Clark County Medical Society 482
Fox River Valley Medical Association 484
Jackson County Medical Society—First Regular Meeting 486

### FOREIGN CORRESPONDENCE.

Treatment for Sea Sickness—Liverpool Hospitals—School for Nurses—English Country Surgeons, Manner of Conducting Business—London—New Anæsthetic in Use—Dangers of Chloroform 487

### SELECTIONS.

Indiscriminate Use of Alcoholic Stimulants in Disease. A Lecture Delivered at Guy's Hospital, London. By Samuel Wilks, M.D., Physician to, and Lecturer on Medicine at the Hospital 492

### BOOK NOTICES.

On Railway and Other Injuries of the Nervous System. By John Eric Erichsen, Fellow of the Royal College of Surgeons; Professor of Surgery and of Clinical Surgery in University College; Surgeon to University College Hospital, etc., etc., etc. Philadelphia: Henry C. Lea. 1867 499

### EDITORIAL.

Chicago Medical Society 500
George K. Amerman, M.D. 502
New College Building 504
Medical Convention in Wisconsin 504
Mortality Report 505
Surgeon J. H. Baxter, of U. S. Volunteers 506
Chlorate of Potassa in Pneumonia 506
Money Receipts 506

